# First molecular detection and genomic characterization of West Nile virus associated with a naturally aborted sheep in Tunisia

**DOI:** 10.1016/j.crpvbd.2026.100432

**Published:** 2026-09-03

**Authors:** Sonia Boughattas, Ahmed Ouni, Kaouthar Ayouni, Takwa Jlassi, Ali Bouattour, Wasfi Fares, Youmna M’ghirbi

**Affiliations:** a Laboratoire Virus Vecteurs et Hôtes: One Health, Institut Pasteur Tunis, Université Tunis El Manar, Tunis, Tunisia; b Departement d’Entomologie Médicale, Institut Pasteur Tunis, Tunis, Tunisia; c Departement de Virologie Clinique, Institut Pasteur Tunis, Tunis, Tunisia

**Keywords:** West Nile virus, Abortion, Ovine, Whole genome sequencing, Oxford nanopore, Tunisia

## Abstract

In September 2025, West Nile virus (WNV) RNA was detected for the first time in a sheep in Zaghouan Governorate, Tunisia. Following sheep abortions, fetal specimens were analyzed and one tested positive for WNV by RT-qPCR. The generated near-complete WNV genome sequence by Oxford Nanopore Technologies belonged to Lineage 1a, previously associated with locally reported human neuroinvasive cases in the same area. Amino acid analysis of the envelope (E) protein confirmed the presence of the conserved NYS glycosylation motif (E154-E156), a characteristic feature of Euro-Mediterranean Lineage 1 WNV strains. These findings further support the endemic circulation of WNV in Tunisia and underline its significance for both animal and public health.

## Highlights

• Abortion cases in an ovine flock were observed in a region with West Nile virus (WNV) occurrence in Tunisia.
• RT-qPCR confirmed the detection of WNV within sheep tissue.
• Oxford Nanopore Technologies enabled near-complete genome sequencing of the virus.

## Graphical abstract

**Figure:**
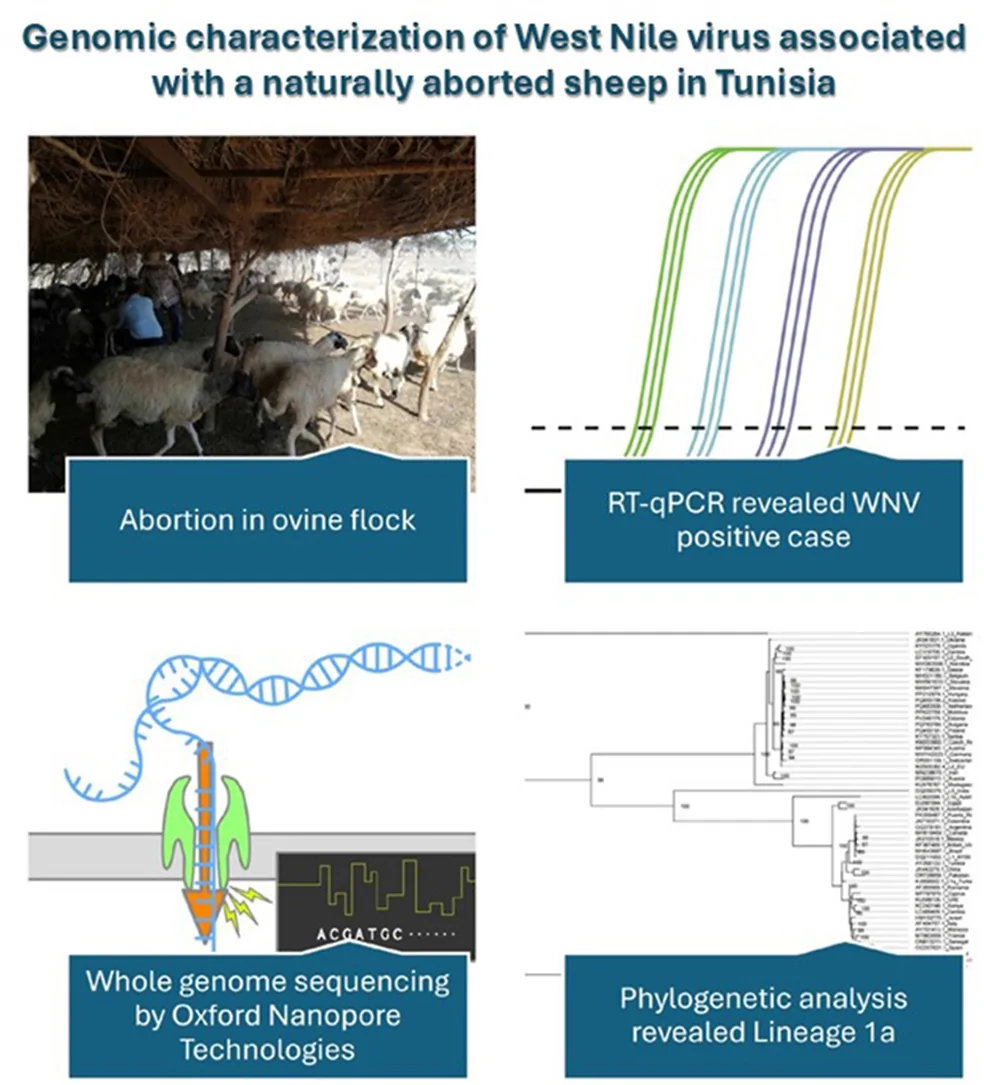

## 1 Introduction

Serological evidence of West Nile virus (WNV), recently classified as *Orthoflavivirus nilense* under the updated binomial species naming system adopted by the International Committee on Taxonomy of Viruses (ICTV), dates back to 1968 in Tunisia (Nabli et al., 1970). However, the first documented human outbreak of WNV occurred in 1997 and was associated with severe neurological disease and deaths among patients (Triki et al., 2001). Subsequent outbreaks were recorded in 2003, 2012, 2018, and 2023, with varying degrees of morbidity and mortality (https://www.onmne.tn/?page_id=7864&lang=en). In parallel to these neuroinvasive cases, evidence of WNV circulation among different hosts was revealed by serological investigations in birds (Hammouda et al., 2015), equines and ruminants (M’ghirbi et al., 2023;Ouni et al., 2025). Viral circulation was confirmed using molecular approaches in both chickens as a sentinel host (Amdouni et al., 2020) and mosquitoes (M’ghirbi et al., 2023; Barhoumi et al., 2025), supporting its endemicity in Tunisia.

However, comprehensive data on the host range of WNV, particularly its impact and pathogenicity in ruminants, remain scarce. This limitation hinders a full understanding of the viral transmission cycle and, consequently, the implementation of effective surveillance strategies. Here, we report the identification and near-full characterization of the whole-genome sequence of WNV detected in tissues associated with sheep abortion using next-generation sequencing.

## 2 Materials and methods

### 2.1 Sampling

As part of a surveillance of pathogens in small ruminants, a farm located in El Fahs (Zaghouan Governorate), northern Tunisia (36°23′N, 9°54′E), was investigated. The investigated flock comprised 24 sheep, including four pregnant ewes that experienced abortion during late gestation in the absence of preceding clinical signs. In the same region, human infections with West Nile virus were documented during the same transmission season (https://www.onmne.tn/?page_id=7864&lang=en). Consequently, the potential involvement of WNV within the affected animals was considered. Accordingly, tissue samples were collected from each of the four aborted fetuses, transported under refrigerated conditions (4 °C) to the Department of Medical Entomology, Pasteur Institute of Tunis, Tunis, Tunisia, for comprehensive laboratory investigation. Total nucleic acids were extracted from tissues using the QIAamp® Cador Pathogen Mini Kit (Qiagen, Hilden, Germany) in accordance with the manufacturer’s protocol, and eluates were stored at −80 °C pending downstream analyses.

### 2.2 Pathogen detection

The extracted nucleic acids were subsequently analyzed by molecular assays targeting major abortifacient pathogens, namely Rift Valley fever virus by RT-qPCR approach (Wilson et al., 2013), *Coxiella burnetii* by nested PCR targeting the *htpB* gene (To et al., 1996) and *Toxoplasma gondii* also by nested PCR targeting the ITS marker (Hurtado et al., 2001). Further, investigation of WNV was performed by running a specific RT-qPCR (Tang et al., 2006; M’ghirbi et al., 2023)) on an Applied Biosystems™ 7500 real-time thermocycler.

### 2.3 WNV next-generation sequencing

The positive WNV RNA was randomly reverse-transcribed to cDNA and subsequently amplified using the Q5 High-Fidelity 2× Master mix DNA Polymerase (New England Biolabs, Ipswich, USA) with two independent primer sets of 11 pairs of primers each designed to generate 22 overlapping amplicons of 600 nucleotides in size, spanning the WNV genome (Ayouni et al., 2026). An input of 50 ng of PCR product was used to attempt whole-genome sequencing. Library preparation was performed following the PCR tiling workflow originally developed for SARS-CoV-2 (ARTIC Network, London, UK) using SQK-NBD114.24 Native Barcoding Kit 24V14 (Oxford Nanopore Technologies, Oxford, UK). A final input of 30 ng of the produced sequencing libraries was then loaded onto R.10 flow cell (FLO-MIN114, Oxford Nanopore Technologies). Sequencing was conducted on an Oxford Nanopore Mk1C sequencer for an 8-h run, with basecalling performed in real time using ONT Guppy (Rapid/Fast basecalling model; software version 6.5.7). The generated Passed FASTQ files were processed and analyzed with the online WNV Genome Detective typing tool (https://www.genomedetective.com/app/typingtool/wnv/) according to the Default Virus Analysis (metagenome) protocol and Pan-viral (2·21·4) adapted to the nanopore sequencing technology.

Multiple sequences alignment was achieved using MAFFT v.7.526 and maximum-likelihood phylogenetic inference was performed with IQ-TREE v.2.0.7 under the best-fit nucleotide substitution model, and node support was assessed using 1000 bootstrap replicates. Moreover, the amino acid sequence of the envelope (E) protein was extracted and analyzed to identify amino acid substitutions potentially associated with WNV virulence in mammals (Fiacre et al., 2020). The deduced E protein sequence was then aligned using ClustalW with the reference sequence of the WNV strain NY99.

## 3 Results

In the present study, no evidence of Rift Valley fever virus, *Coxiella burnetii*, and *Toxoplasma gondii* pathogens was detected in any of the tested samples. However, WNV RNA was detected in one fetal tissue sample collected from an abortion case with RT-qPCR positivity at Ct = 32.80.

The subsequent ONT sequencing generated a total of 1,029,924 raw nanopore sequencing reads (read length range: 65–90,260 bp) from the investigated sample. Following quality control and filtering, 42,408 reads (4.1%) were retained for downstream analysis. Of these, 1972 reads (0.2% of the total dataset) were assigned to the West Nile virus candidate reference sequence, and 1889 high-quality reads were used for consensus sequence generation after additional filtering and primer trimming. The final consensus sequence was subsequently validated by remapping all reads against the assembled genome, with 67,345 reads (6.5% of the total dataset) aligning to the reconstructed contigs. Despite the relatively small proportion of virus-specific reads, the targeted amplicon sequencing approach enabled the reconstruction of a near-complete WNV genome (10,347 nt, 95.3% genome coverage) with an average sequencing depth of 71×.

**Fig. 1:**
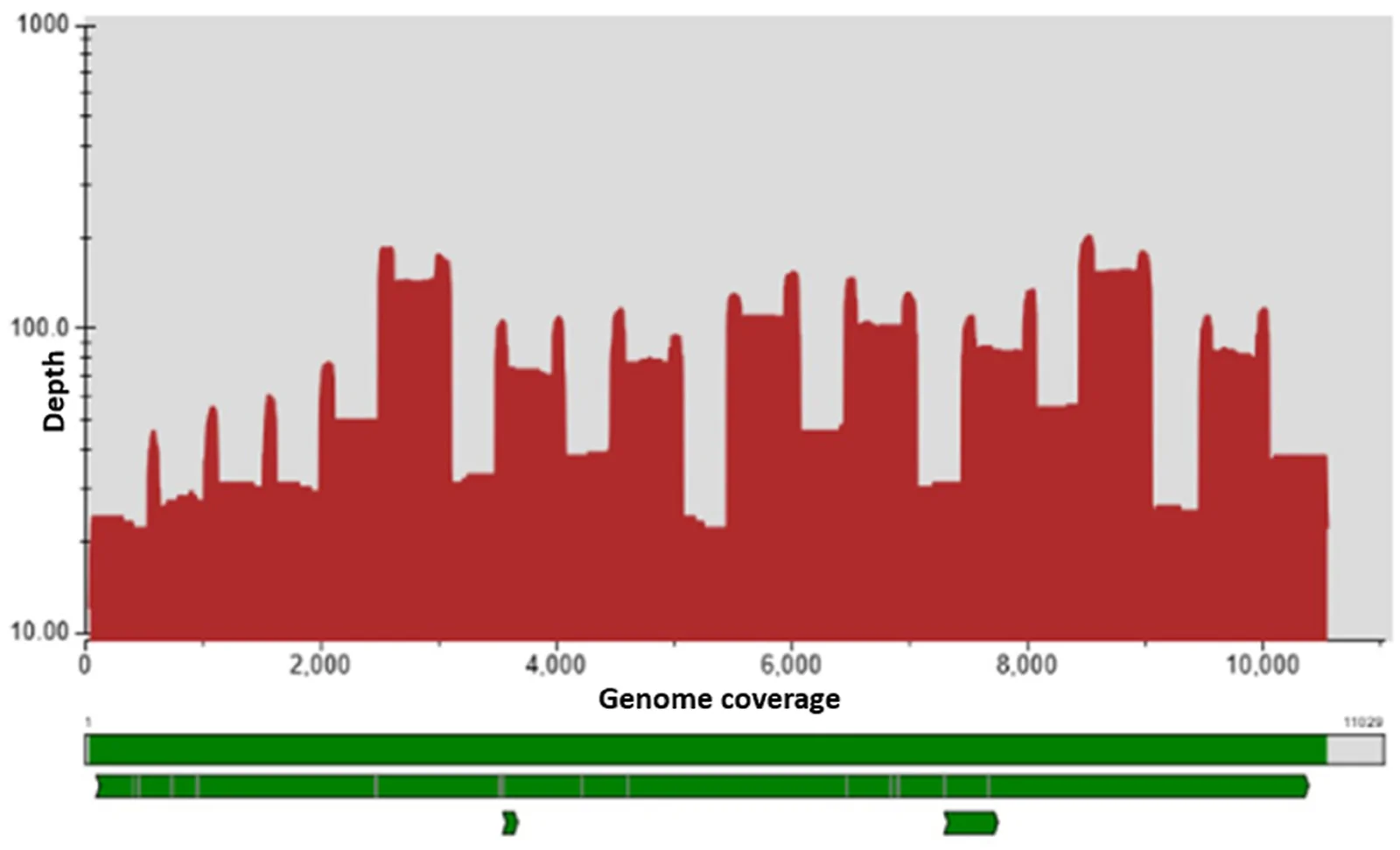
Coverage map of the generated WNV whole-genome sequence.

After a BLASTN step, the obtained sequence exhibited 98.9% nucleotide identity across 100% query coverage with the WNV isolate SN2016-286301, which was recovered from *Culex poicilipes* mosquitoes collected in Senegal in 2016 (GenBank: ON813211.1). The assembled genome covered the entire coding sequence (CDS), encoding the viral polyprotein (3434AA) together with two additional truncated proteins, the WARF4 open reading frame (2554AA) product and the truncated NS1′ protein (1196AA), which are known features of WNV Lineage 1a viruses (Fig. 1).

**Fig. 2:**
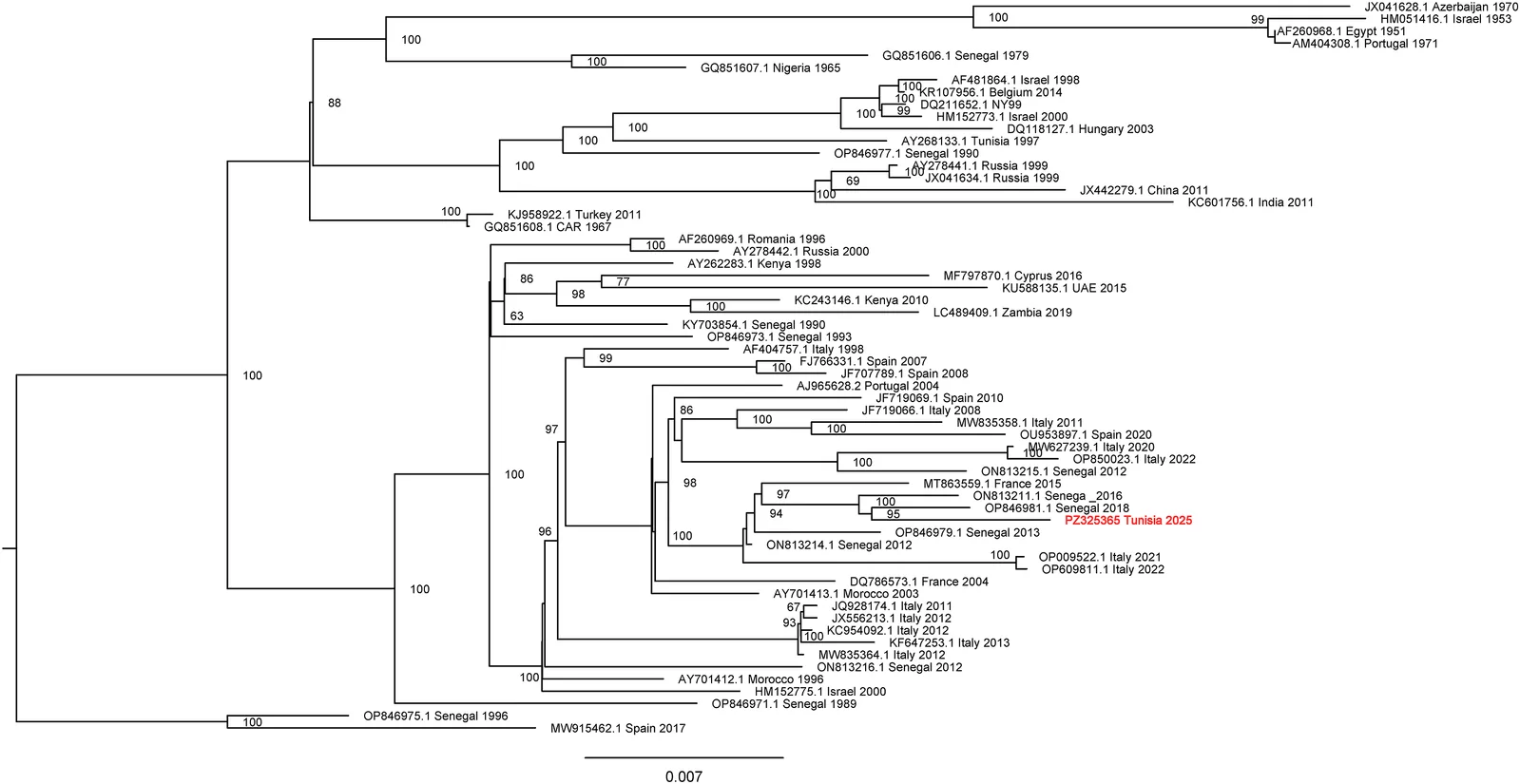
Maximum likelihood phylogenetic tree for WNV Lineage 1a according to the model GTR+F+I+G4 showing the position of the local WNV strain (represented in red).

**Fig. 3:**
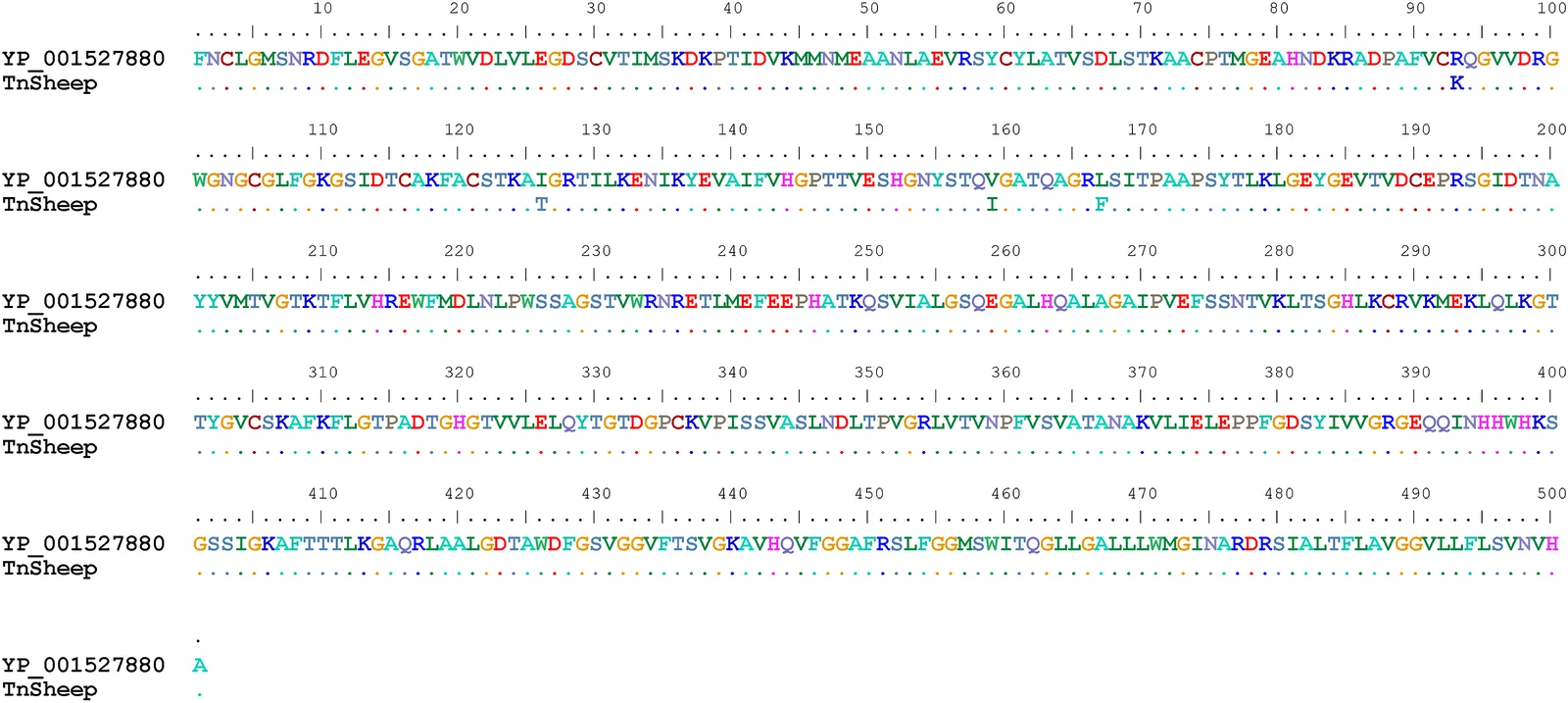
Amino-acid sequence alignment of the E gene product. Dots (.) indicate identical AA related to the reference sequence for the WNV NY99 (YP_001527880) strain in the first row.

The retrieved consensus WNV sequence was aligned with a panel of 59 WNV whole-genome sequences of Lineage 1a covering broad geographical distribution (Table 1). Phylogenetic analysis (Fig. 2) assigned the newly characterized Tunisian WNV strain to Lineage 1a, cluster C2, within the Western Mediterranean (WMED1) subcluster. Our sequence clustered with strong bootstrap support (> 95%) with the Senegalese isolates identified in 2016 and 2018 and grouped within a well-supported clade containing strains circulating across West Africa (namely Senegal) and the Mediterranean Basin, including isolates from Portugal, Spain, France, Morocco and Italy. Members of this clade have been recovered from diverse hosts, including birds, mosquitoes, equids, and humans, highlighting the close genetic relationship between the Tunisian strain and Lineage 1a WNVs characterized by north-western African origin, with back-and-forth exchanges between West Africa and southern-western Europe (Mencattelli et al., 2023; Aguilera-Sepúlveda et al., 2024). Furthermore, as part of the molecular characterization of the virus, the isolate’s amino-acid sequence analysis of the envelope (E) gene, revealed the presence of the conserved NYS glycosylation motif (E154-E156) without the I159V mutation, a characteristic feature of Euro-Mediterranean Lineage 1 WNV strains (Fig. 3).

**Table 1:** List of the WNV Lineage 1a sequences retrieved from Mencattelli et al. (2023) and used in the phylogenetic analysis.

GenBank ID	Country	Year	Host	Sub-lineage
AF260968.1	Egypt	1951	*Homo sapiens*	C1
AF260969.1	Romania	1996	*Culex pipiens*	C2/EEC
AF404757.1	Italy	1998	*Equus caballus*	C2/WMED2
AF481864.1	Israel	1998	*Ciconia ciconia*	C4
AJ965628.2	Portugal	2004	*Cx. pipiens + Cx. univittatus*	C2/WMED1
AM404308.1	Portugal	1971	*Anophleles maculipennis*	C1
AY262283.1	Kenya	1998	*Culex univittatus*	C2
AY268133.1	Tunisia	1997	*Homo sapiens*	C4
AY278441.1	Russia	1999	*Homo sapiens*	C2/EEC
AY278442.1	Russia	2000	*Homo sapiens*	C2/EEC
AY701412.1	Morocco	1996	*Equus caballus*	C2/WMED
AY701413.1	Morocco	2003	*Equus caballus*	C2/WMED1
DQ118127.1	Hungary	2003	*Anser anser*	C4
DQ211652.1	USA	1999	*Corvus brachyrhynchos*	C4
DQ786573.1	France	2004	*Pica pica*	C2/WMED1
FJ766331.1	Spain	2007	*Aquila chrysaetos*	C2/WMED2
GQ851606.1	Senegal	1979	Arthropod vector	C5
GQ851607.1	Nigeria	1965	Unkown	C5
GQ851608.1	Central African Republic	1967	Unkown	C6
HM051416.1	Israel	1953	*Homo sapiens*	C1
HM152773.1	Israel	2000	*Homo sapiens*	C4
HM152775.1	Israel	2000	*Homo sapiens*	C2/WMED
JF707789.1	Spain	2008	*Culex perexiguus*	C2/WMED2
JF719066.1	Italy	2008	*Pica pica*	C2/WMED1
JF719069.1	Spain	2010	*Equus caballus*	C2/WMED1
JQ928174.1	Italy	2011	*Homo sapiens*	C2/LIVENZA
JX041628.1	Azerbaijan	1970	*Ornithodoros capensis*	C1
JX041634.1	Russia	1999	*Homo sapiens*	C3
JX442279.1	China	2011	*Culex pipiens*	C3
JX556213.1	Italy	2012	*Homo sapiens*	C2/LIVENZA
KC243146.1	Kenya	2010	*Rhipicephalus pulchellus*	C2
KC601756.1	India	2011	*Homo sapiens*	C3
KC954092.1	Italy	2012	*Homo sapiens*	C2/LIVENZA
KF647253.1	Italy	2013	*Homo sapiens*	C2/LIVENZA
KJ958922.1	Turkey	2011	*Equus caballus*	C6
KR107956.1	Belgium	2014	Vero cells	C4
KU588135.1	UAE	2015	*Camelus dromedarius*	C2
KY703854.1	Senegal	1990	*Culex poicilipes*	C2
LC489409.1	Zambia	2019	*Crocodylus niloticus*	C2
MF797870.1	Cyprus	2016	*Homo sapiens*	C2
MT863559.1	France	2015	*Equus caballus*	C2/WMED1
MW627239.1	Italy	2020	*Accipiter gentilis*	C2/WMED1
MW835358.1	Italy	2011	*Corvus corone*	C2/WMED1
MW835364.1	Italy	2012	*Pica pica*	C2/LIVENZA
MW915462.1	Spain	2017	*Accipiter gentilis*	C7
ON813211.1	Senegal	2016	*Culex poicilipes*	C2/WMED1
ON813214.1	Senegal	2012	*Culex neavei*	C2/WMED1
ON813215.1	Senegal	2012	*Culex perfuscus*	C2/WMED1
ON813216.1	Senegal	2012	*Culex quinquefasciatus*	C2/WMED
OP009522.1	Italy	2021	*Culex pipiens*	C2/WMED1
OP609811.1	Italy	2022	*Equus caballus*	C2/WMED1
OP846971.1	Senegal	1989	*Culex poicilipes*	C2
OP846973.1	Senegal	1993	*Culex neavei*	C2
OP846975.1	Senegal	1996	*Aedes dalzieli*	C7
OP846977.1	Senegal	1990	*Culex antennatus*	C4
OP846979.1	Senegal	2013	*Culex neavei*	C2/WMED1
OP846981.1	Senegal	2018	*Homo sapiens*	C2/WMED1
OP850023.1	Italy	2022	*Equus caballus*	C2/WMED1
OU953897.1	Spain	2020	*Homo sapiens*	C2/WMED1

## 4 Discussion

To the best of our knowledge, this is the first report of WNV associated with natural mammal abortion case. According to World Organization for Animal Health, WNV is mainly associated with equines and some birds’ neurological disease with wide range of symptoms going from weakness, stumbling, partial paralysis, impaired vision, convulsions, circling, to coma and death (https://www.woah.org/en/blog/disease/west-nile-fever/). Sheep are generally considered as incidental host, and only few studies have investigated their susceptibility to WNV using serological approaches. Seroprevalence rates ranging from 0.38% to 6.50% have been reported in Tunisia (Khamassi Khbou et al., 2020; Ouni et al., 2025), compared to 1.0% in Slovakia (Juricová et al., 1986), 3.0% in Croatia (Barbic et al., 2025), 3.5% in Egypt (Selim and Abdelhady, 2020), 14.7% in Palestine (Azmi et al., 2017), 25.7% in Ethiopia (Megenas et al., 2025), and up to 58% in Puerto Rico (Phoutrides et al., 2011). Additionally, rare clinical cases involving neurological disorders and encephalitis have been described in sheep in several regions (Tyler et al., 2003; Yaeger et al., 2004; Kecskeméti et al., 2007; Rimoldi et al., 2017). However, an experimental study, conducted in South Africa, investigating inoculation with different flaviviruses, demonstrated that infection with WNV led to abortion in 12.5% of pregnant ewes and was associated with detectable viremia (Barnard and Voges, 1986).

Together with such experimental observations reported by Barnard and Voges (1986), our findings indicate that WNV can be detected in fetal tissues following an abortion event. However, the detection of WNV RNA alone is insufficient to establish a causal relationship between WNV infection and abortion under natural field conditions. Consequently, the present report supports an association between WNV detection and the abortion event, but cannot establish causality. The genomic characterization of the detected WNV revealed clustering within Lineage 1a, aligning with previous results generated by partial Sanger gene sequencing identified in human neuroinvasive cases during the last outbreak in Tunisia (Barhoumi et al., 2025). Moreover, the near-complete whole-genome sequence is also similar to strains of WNV identified in local mosquitoes (our unpublished data). Lineage 1a is known to originate from Senegal before its spread to Europe through different birds’ flyways and its re-introduction to Africa (Ndione et al., 2022).

## 5 Conclusions

Further epidemiological, experimental and genomic explorations are required to elucidate the ability of this neurotropic pathogen to cross the feto-placental barrier, characterize the genomic determinants associated with its virulence, and assess its potential impact on livestock and public health. In addition, identifying the migratory birds’ flyways involved in WNV dissemination would provide valuable insights for strengthening surveillance programmes and informing evidence-based policies for virus monitoring and early detection.

## Ethical approval

The study was conducted according to the guidelines of the Declaration of Helsinki and approved by the Commission on Ethics and Animal Welfare of the Institut Pasteur de Tunis, Tunisia (Protocol Code, 2025/13/I/V1).

## CRediT authorship contribution statement

**Sonia Boughattas:** Formal analysis, Writing - original draft, Writing - review & editing. **Ahmed Ouni:** Methodology. **Kaouthar Ayouni:** Methodology. **Takwa Jlassi:** Methodology. **Ali Bouattour:** Resources, Validation, Writing - review & editing. **Wasfi Fares:** Data curation, Writing - review & editing. **Youmna M’ghirbi:** Conceptualization, Supervision, Writing - review & editing, Funding acquisition.

## Data availability

The generated near-complete WNV whole-genome sequence was submitted to the GenBank database under the accession number PZ325365.

## Statement on the use of AI-assisted technologies

The authors did not use AI-assisted technologies in the manuscript generation process.

## Funding

This study was funded by the Laboratory of Viruses, Vectors and Hosts (LR20IPT02) funded by the Ministry of Higher Education and Scientific Research of Tunisia and the project “Harnessing sequencing-based technologies for pandemic preparedness using One Health-based approach in Tunisia and neighboring countries (SPOT)” (Project No. D82067) funded by the Federal Ministry of Health of Germany through the Global Health Protection Programme (GHPP).

## Declaration of competing interests

The authors declare that they have no known competing financial interests or personal relationships that could have appeared to influence the work reported in this paper.
